# Radiation damage and the case for unpatterned fixed targets

**DOI:** 10.1107/S2052252525011418

**Published:** 2026-01-01

**Authors:** Dominik Oberthür

**Affiliations:** a Center for Free-Electron Laser Science CFEL Deutsches Elektronen-Synchrotron DESY Notkestr. 85 22607Hamburg Germany

**Keywords:** radiation damage, serial femtosecond crystallography, SFX, serial synchrotron crystallography, SSX, diffract-before-destroy, sheet-on-sheet targets

## Abstract

Advances in serial crystallography have reshaped approaches to radiation damage at room temperature. This commentary highlights recent work comparing XFEL and synchrotron experiments, emphasizing its implications for serial crystallography at modern X-ray sources.

Radiation damage arising from the exposure of protein crystals to X-rays has been observed, investigated and discussed since before the first protein structures were solved by X-ray crystallography (Traub & Hirshfeld, 1960 ▸). Over more than 65 years of studying this phenomenon, much has been learned and numerous strategies to minimize radiation damage have been established (Garman, 2010 ▸; Garman & Weik, 2023 ▸). These include cryogenic data collection at the boiling points of liquid nitrogen or helium, the use of higher X-ray energies, distributing the dose across multiple crystals and optimizing beam parameters (*e.g.* employing top-hat beam profiles) to achieve homogeneous illumination of the crystal volume.

For roughly the past 16 years, serial femtosecond crystallography (SFX) (Henkel & Oberthür, 2024 ▸; Chapman *et al.*, 2011 ▸) has provided new opportunities for data collection with virtually no radiation damage (Nass, 2019 ▸; de la Mora *et al.*, 2020 ▸). At X-ray free-electron lasers, ultrashort pulses in the single- to double-digit femtosecond range enable the collection of diffraction data before radiation damage processes begin (Fig. 1 ▸), an approach widely known as ‘diffraction before destruction’ (Chapman *et al.*, 2014 ▸). Serial synchrotron crystallography (SSX) (Stellato *et al.*, 2014 ▸) on the other hand is mostly benefitting from pushing the distribution of dose across multiple crystals to the extreme: each crystal is only exposed once. Using polychromatic X-rays for SSX (Meents *et al.*, 2017 ▸), the exposure time per crystal can be in the range of a few hundred picoseconds, which is still far away from ‘diffract-before-destruct’, but secondary damage based on the diffusion of radicals could be mitigated using this approach. Still, radiation damage in SSX remains poorly understood and current models of radiation damage do not fully explain experimental observations. In a room-temperature SSX study of *Aspergillus flavus* urate oxidase in complex with 5PMUA, no ‘obvious signs of 5PMUA radiolysis’ could be detected, whereas at similar doses under cryo-conditions clear signs of radiolysis could be detected (Zielinski *et al.*, 2022 ▸).

In their article *Testing the limits: serial crystallography using unpatterned fixed targets* (Gorel *et al.*, 2025 ▸), published in the November 2025 issue of *IUCrJ*, the authors systematically investigate and compare radiation damage in SFX and SSX using a single protein system (the heme protein dye-decolourizing peroxidase DtpAa from *Streptomyces lividans*) and an identical sample-delivery approach at both SwissFEL and the ESRF. The paper is not an easy read, its analyses are dense and, at first glance, even somewhat disorienting. Yet the effort is well rewarded: a careful reading yields a deeper insight into radiation damage, practical guidance for planning experiments at both XFELs and synchrotrons, and indications of where future work might further clarify damage mechanisms. It also serves as a cautionary reminder of the importance of scrutinizing data and analysing experimental results with care.

What distinguishes the study by Gorel *et al.* from many earlier investigations is the degree of experimental control achieved. By using the same protein, identical crystal preparation and unpatterned sheet-on-sheet fixed targets at both SwissFEL and the ESRF, the authors minimize confounding factors related to sample handling and delivery. This allows differences between the datasets to be attributed primarily to the X-ray pulse structure and exposure timescale, rather than to changes in geometry or sample environment.

The SFX data collected at SwissFEL largely conform to established expectations. For step sizes between exposures of 25 µm and above, diffraction quality remains stable and sensitive structural parameters of the heme active site show no systematic trends, consistent with radiation damage remaining below detectable levels. Only when exposure positions are brought into closer proximity do changes emerge, including a loss of high-resolution diffraction and alterations in unit-cell parameters. These effects are consistent with indirect damage mechanisms, such as diffusion or dehydration, rather than direct X-ray-induced chemistry during the femtosecond pulse itself.

The serial synchrotron crystallography data reveal a more nuanced picture. Although each crystal is exposed only once, using a single 90 µs exposure, the diffraction data show clear signatures consistent with global radiation damage developing during the exposure itself. Compared with the XFEL measurements, the SSX data exhibit a steeper fall-off of high-resolution intensities and elevated Wilson *B* factors, indicative of increased lattice disorder. At the same time, subtle but reproducible changes in heme geometry suggest that local radiation-induced effects accompany this global degradation, even though the distal water ligand remains structurally intact on this timescale.

A key aspect of this interpretation concerns the role of transient heating during the synchrotron exposure. Using a previously described model (Warren *et al.*, 2019 ▸), the authors estimate that the equilibrium temperature rise of a DtpAa crystal exposed at beamline ID29 (ESRF) could, in principle, exceed 1500 K. They are careful to stress that this value is almost certainly a strong overestimate, reflecting known limitations of the model. Nevertheless, given the high absorbed dose delivered within a single 90 µs exposure, a substantial temperature increase during irradiation appears unavoidable. Such heating would not necessarily lead to macroscopic crystal damage, indeed, post-exposure optical microscopy reveals no obvious changes in crystal morphology, but disordering on submicrometre length scales would remain invisible by this method.

It is therefore conceivable that transient temperature rises induce molecular motion within the crystal lattice, leading to a loss of long-range order while leaving the overall crystal intact. In diffraction terms, this scenario would manifest as a redistribution of Bragg intensities from high to low resolution and an increase in the Wilson *B* factor, a hallmark of Bragg termination (Lomb *et al.*, 2011 ▸; Barty *et al.*, 2012 ▸). Consistent with this picture, the ID29 data show systematically higher Wilson *B* factors than the XFEL data, independent of crystal batch or experiment date. The authors also compared Wilson plots calculated from refined model intensities, which avoid complications arising from background and scaling and could show that the difference becomes particularly evident in this comparison. Taken together, these observations strongly suggest that global disorder develops during the synchrotron exposure itself, highlighting transient heating as an important contributor to radiation-induced damage at fourth-generation sources (Fig. 1 ▸).

An important practical insight emerging from the study concerns the use of unpatterned fixed targets. Unlike microfabricated chips with physical barriers between crystals, sheet-on-sheet supports allow heat, radicals or gas generated by previous exposures to propagate freely. While this makes experiments more susceptible to indirect damage, it also turns such targets into a sensitive probe for radiation effects that might otherwise remain obscured. Moreover the work by Gorel *et al.* shows that those chips can be used for SFX at XFELs without inducing further radiation damage if step sizes larger than 25 µm between exposures are used.

Taken together, the results challenge the assumption that ‘one exposure per crystal’ is necessarily sufficient to avoid meaningful radiation damage in room-temperature SSX experiments at modern high-flux beamlines. Instead, they highlight that damage may arise on microsecond timescales within a single still image, underscoring the need to consider not only absorbed dose but also dose rate and exposure duration. As serial crystallography continues to expand across both XFELs and fourth-generation synchrotrons, studies such as this provide a timely reminder that radiation damage remains a moving target, one that demands careful experimental design, critical data analysis and continued refinement of damage models.

## Figures and Tables

**Figure 1:**
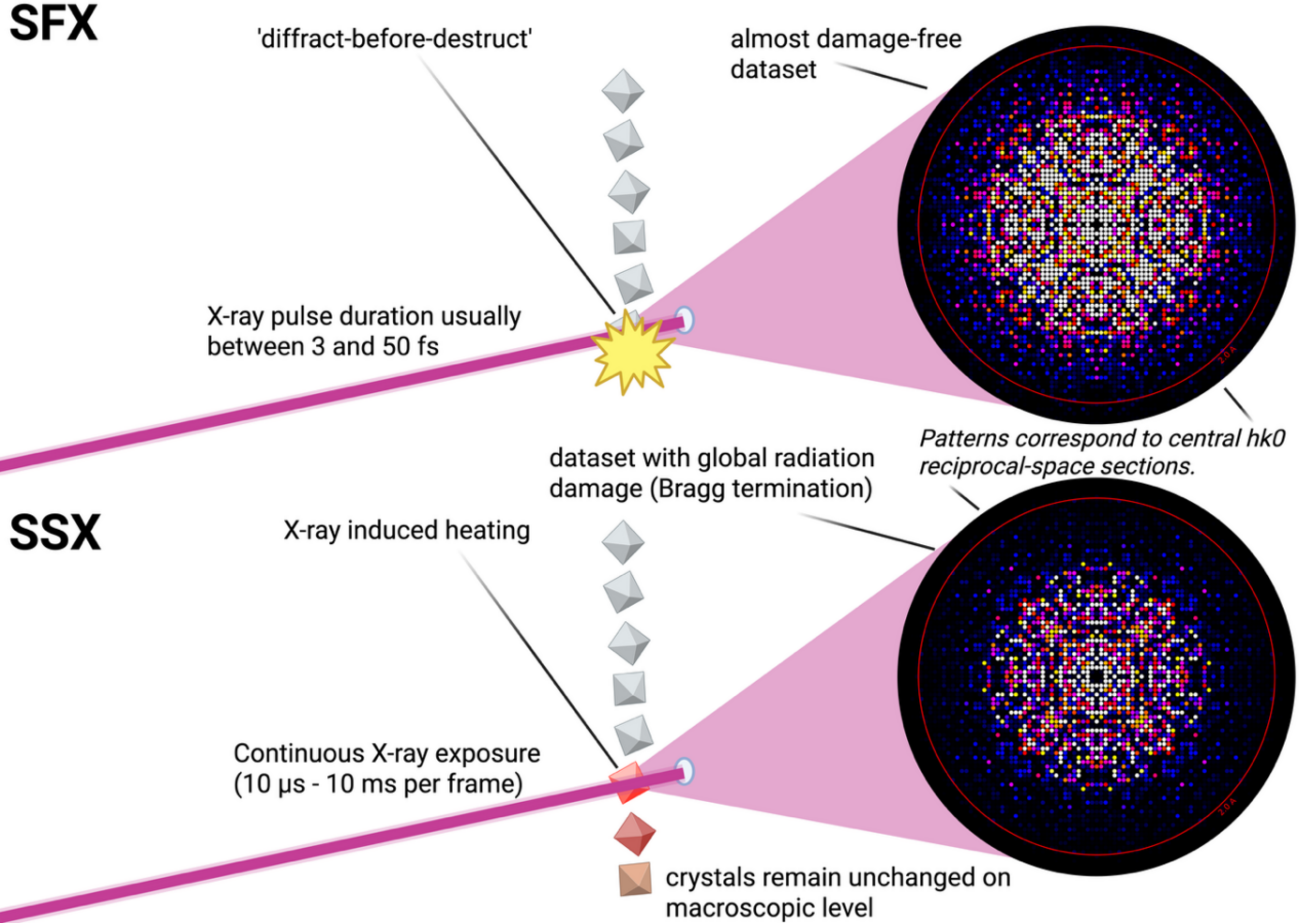
Schematic comparison of serial femtosecond crystallography (SFX) and serial synchrotron crystallography (SSX). In SFX (top), femtosecond X-ray pulses enable diffraction before radiation damage develops, yielding largely damage-free datasets. In SSX (bottom), longer exposures can induce transient heating and molecular motion during irradiation, leading to global lattice disorder and attenuation of high-resolution Bragg intensities (Bragg termination). The two-dimensional plots of central *hk*0 sections of the reciprocal lattice were generated using *render_hkl* from *CrystFEL* (White *et al.*, 2012 ▸) for representative SFX and SSX datasets, respectively; their use here is purely schematic. Created in *bioRender* (https://biorender.com).

## References

[bb1] Barty, A., Caleman, C., Aquila, A., Timneanu, N., Lomb, L., White, T. A., Andreasson, J., Arnlund, D., Bajt, S., Barends, T. R. M., Barthelmess, M., Bogan, M. J., Bostedt, C., Bozek, J. D., Coffee, R., Coppola, N., Davidsson, J., DePonte, D. P., Doak, R. B., Ekeberg, T., Elser, V., Epp, S. W., Erk, B., Fleckenstein, H., Foucar, L., Fromme, P., Graafsma, H., Gumprecht, L., Hajdu, J., Hampton, C. Y., Hartmann, R., Hartmann, A., Hauser, G., Hirsemann, H., Holl, P., Hunter, M. S., Johansson, L., Kassemeyer, S., Kimmel, N., Kirian, R. A., Liang, M., Maia, F. R. N. C., Malmerberg, E., Marchesini, S., Martin, A. V., Nass, K., Neutze, R., Reich, C., Rolles, D., Rudek, B., Rudenko, A., Scott, H., Schlichting, I., Schulz, J., Seibert, M. M., Shoeman, R. L., Sierra, R. G., Soltau, H., Spence, J. C. H., Stellato, F., Stern, S., Strüder, L., Ullrich, J., Wang, X., Weidenspointner, G., Weierstall, U., Wunderer, C. B. & Chapman, H. N. (2012). *Nat. Photon.* **6**, 35–40.

[bb2] Chapman, H. N., Caleman, C. & Timneanu, N. (2014). *Phil. Trans. R. Soc. B* **369**, 20130313. 10.1098/rstb.2013.0313 PMC4052855 24914146

[bb3] Chapman, H. N., Fromme, P., Barty, A., White, T. A., Kirian, R. A., Aquila, A., Hunter, M. S., Schulz, J., DePonte, D. P., Weierstall, U., Doak, R. B., Maia, F. R. N. C., Martin, A. V., Schlichting, I., Lomb, L., Coppola, N., Shoeman, R. L., Epp, S. W., Hartmann, R., Rolles, D., Rudenko, A., Foucar, L., Kimmel, N., Weidenspointner, G., Holl, P., Liang, M., Barthelmess, M., Caleman, C., Boutet, S., Bogan, M. J., Krzywinski, J., Bostedt, C., Bajt, S., Gumprecht, L., Rudek, B., Erk, B., Schmidt, C., Hömke, A., Reich, C., Pietschner, D., Strüder, L., Hauser, G., Gorke, H., Ullrich, J., Herrmann, S., Schaller, G., Schopper, F., Soltau, H., Kühnel, K.-U., Messerschmidt, M., Bozek, J. D., Hau-Riege, S. P., Frank, M., Hampton, C. Y., Sierra, R. G., Starodub, D., Williams, G. J., Hajdu, J., Timneanu, N., Seibert, M. M., Andreasson, J., Rocker, A., Jönsson, O., Svenda, M., Stern, S., Nass, K., Andritschke, R., Schröter, C.-D., Krasniqi, F., Bott, M., Schmidt, K. E., Wang, X., Grotjohann, I., Holton, J. M., Barends, T. R. M., Neutze, R., Marchesini, S., Fromme, R., Schorb, S., Rupp, D., Adolph, M., Gorkhover, T., Andersson, I., Hirsemann, H., Potdevin, G., Graafsma, H., Nilsson, B. & Spence, J. C. H. (2011). *Nature* **470**, 73–77.

[bb4] de la Mora, E., Coquelle, N., Bury, C. S., Rosenthal, M., Holton, J. M., Carmichael, I., Garman, E. F., Burghammer, M., Colletier, J.-P. & Weik, M. (2020). *Proc. Natl Acad. Sci.* **117**, 4142–4151. 10.1073/pnas.1821522117 PMC7049125 32047034

[bb5] Garman, E. F. (2010). *Acta Cryst.* D**66**, 339–351. 10.1107/S0907444910008656 PMC2852297 20382986

[bb6] Garman, E. F. & Weik, M. (2023). *Curr. Opin. Struct. Biol.* **82**, 102662. 10.1016/j.sbi.2023.102662 37573816

[bb7] Gorel, A., Shoeman, R. L., Hartmann, E., Nizinski, S., Appleby, M. V., Beale, E. V., Dworkowski, F., Gotthard, G. C., Beale, J. H., Holton, J., Doak, R. B., Barends, T. R. M. & Schlichting, I. (2025). *IUCrJ* **12**, 692–709. 10.1107/S2052252525008371 PMC12573926 41081786

[bb8] Henkel, A. & Oberthür, D. (2024). *Acta Cryst.* D**80**, 563–579. 10.1107/S2059798324005588 PMC11301758 38984902

[bb9] Lomb, L., Barends, T. R. M., Kassemeyer, S., Aquila, A., Epp, S. W., Erk, B., Foucar, L., Hartmann, R., Rudek, B., Rolles, D., Rudenko, A., Shoeman, R. L., Andreasson, J., Bajt, S., Barthelmess, M., Barty, A., Bogan, M. J., Bostedt, C., Bozek, J. D., Caleman, C., Coffee, R., Coppola, N., DePonte, D. P., Doak, R. B., Ekeberg, T., Fleckenstein, H., Fromme, P., Gebhardt, M., Graafsma, H., Gumprecht, L., Hampton, C. Y., Hartmann, A., Hauser, G., Hirsemann, H., Holl, P., Holton, J. M., Hunter, M. S., Kabsch, W., Kimmel, N., Kirian, R. A., Liang, M., Maia, F. R. N. C., Meinhart, A., Marchesini, S., Martin, A. V., Nass, K., Reich, C., Schulz, J., Seibert, M. M., Sierra, R., Soltau, H., Spence, J. C. H., Steinbrener, J., Stellato, F., Stern, S., Timneanu, N., Wang, X., Weidenspointner, G., Weierstall, U., White, T. A., Wunderer, C., Chapman, H. N., Ullrich, J., Strüder, L. & Schlichting, I. (2011). *Phys. Rev. B* **84**, 214111.

[bb10] Meents, A., Wiedorn, M. O., Srajer, V., Henning, R., Sarrou, I., Bergtholdt, J., Barthelmess, M., Reinke, P. Y. A., Dierksmeyer, D., Tolstikova, A., Schaible, S., Messerschmidt, M., Ogata, C. M., Kissick, D. J., Taft, M. H., Manstein, D. J., Lieske, J., Oberthuer, D., Fischetti, R. F. & Chapman, H. N. (2017). *Nat. Commun.* **8**, 1281. 10.1038/s41467-017-01417-3 PMC5668288 29097720

[bb11] Nass, K. (2019). *Acta Cryst.* D**75**, 211–218. 10.1107/S2059798319000317 PMC6400258 30821709

[bb12] Stellato, F., Oberthür, D., Liang, M., Bean, R., Gati, C., Yefanov, O., Barty, A., Burkhardt, A., Fischer, P., Galli, L., Kirian, R. A., Meyer, J., Panneerselvam, S., Yoon, C. H., Chervinskii, F., Speller, E., White, T. A., Betzel, C., Meents, A. & Chapman, H. N. (2014). *IUCrJ* **1**, 204–212. 10.1107/S2052252514010070 PMC4107920 25075341

[bb13] Traub, W. & Hirshfeld, F. L. (1960). *Acta Cryst.* **13**, 753–760.

[bb14] Warren, A. J., Axford, D. & Owen, R. L. (2019). *J. Synchrotron Rad.* **26**, 991–997. 10.1107/S1600577519003849 PMC6613110 31274420

[bb15] White, T. A., Kirian, R. A., Martin, A. V., Aquila, A., Nass, K., Barty, A. & Chapman, H. N. (2012). *J. Appl. Cryst.* **45**, 335–341.

[bb16] Zielinski, K. A., Prester, A., Andaleeb, H., Bui, S., Yefanov, O., Catapano, L., Henkel, A., Wiedorn, M. O., Lorbeer, O., Crosas, E., Meyer, J., Mariani, V., Domaracky, M., White, T. A., Fleckenstein, H., Sarrou, I., Werner, N., Betzel, C., Rohde, H., Aepfelbacher, M., Chapman, H. N., Perbandt, M., Steiner, R. A. & Oberthuer, D. (2022). *IUCrJ* **9**, 778–791. 10.1107/S2052252522010193 PMC9634612 36381150

